# TiO_2_ Nanotube Array Sensor for Detecting the SF_6_ Decomposition Product SO_2_

**DOI:** 10.3390/s120303302

**Published:** 2012-03-07

**Authors:** Xiaoxing Zhang, Jinbin Zhang, Yichao Jia, Peng Xiao, Ju Tang

**Affiliations:** 1 State Key Laboratory of Power Transmission Equipment & System Security and New Technology, Chongqing University, Chongqing 400044, China; E-Mails: zhangjinbin023@126.com (J.Z.); cqtang@vip.sina.com (J.T.); 2 College of Mathematics and Physics, Chongqing University, Chongqing 400044, China; E-Mails: cailing123456@yahoo.cn (Y.J.); huanglinspecial@hotmail.com (P.X.)

**Keywords:** TiO_2_ nanotube array, SF_6_ decomposed components, SO_2_ gas, sensor response

## Abstract

The detection of partial discharge through analysis of SF_6_ gas components in gas-insulated switchgear, is significant for the diagnosis and assessment of the operating state of power equipment. The present study proposes the use of a TiO_2_ nanotube array sensor for detecting the SF_6_ decomposition product SO_2_, and the application of the anodic oxidation method for the directional growth of highly ordered TiO_2_ nanotube arrays. The sensor response of 10–50 ppm SO_2_ gas is tested, and the sensitive response mechanism is discussed. The test results show that the TiO_2_ nanotube sensor array has good response to SO_2_ gas, and by ultraviolet radiation, the sensor can remove attached components very efficiently, shorten recovery time, reduce chemical poisoning, and prolong the life of the components.

## 1. Introduction

SF_6_ has been widely used in gas-insulated switchgear (GIS) because it has good insulating performance and arc extinction, and it can markedly improve insulation intensity [1–4] when used as an insulating medium. Although the reliability of GIS equipment is very high, inevitable failures due to internal defects can still cause different degrees of partial discharge. The active gas generated by discharging electricity accelerates insulation aging and corrodes the metal surface, which may eventually trigger a GIS fault. A massive amount of research locally and abroad demonstrates that when insulation faults occur in GIS, discharging electricity energy causes the SF_6_ gas to undergo a decomposition reaction, thus producing several kinds of low-fluorine sulfides, such as SF_4_, SF_3_, SF_2_, and so on. These low-fluorine sulfides react further with trace moisture and oxygen in the SF_6_ gas, thus producing the compounds SOF_4_, SOF_2_, SO_2_F_2_, SO_2_, and HF, among others [5,6]. At present, the main methods used to analyze and detect the decomposition component of SF_6_ partial discharge are gas chromatography, test tube method, infrared absorption spectrometry, and so on. Consequently, there has been increasing interest in the use of the contents of the characteristic components of the gas detect GIS internal insulation faults.

Titanium dioxide nanotube array (TiO_2_ NTs) is a typical three-dimensional nanomaterial. TiO_2_ NTs has rich chemical and physical properties and low manufacturing costs. Thus, TiO_2_ NTs has broad application prospects [7]. In recent years, research has shown that because of its large specific surface area and nanosize effect, the TiO_2_ nanotube arrays have an enormous potential for development compared with other nanostructure forms in fields such as light catalysis, sensor, and solar batteries. TiO_2_ nanotube arrays have become the hotspot of international nanometer material research [8]. The tiny gas sensor made from TiO_2_ NTs has several advantages, such as fast response, high sensitivity, and small size. Several scholars in the field have achieved significant progress. As a sensitive material, TiO_2_ NTs is used to test O_2_, NO_2_, H_2_, ethanol, and other gases [9–14]. Some key references about TiO_2_ nanotube sensors in the existing literature are listed below in Table 1:

**Table 1.** The research situation of TiO_2_ nanotubes sensor.

**Available Literature**	**Nanotube Type**	**Nanotube diameter**	**Gas sensor**	**Sensitivity**
Seo, M.-H., *et al*. [10]	TiO_2_ individual nanotubes	70 nm	Toluene (50 ppm)	25%
Lin, S., *et al*. [11]	TiO_2_ nanotube arrays	150 nm	Formaldehyde (50 ppm)	35%
Yun, H. [12]	TiO_2_ individual nanotubes	20 nm	NO_2_ (12.5 ppm)	67%
	TiO_2_ nanotube arrays	100 nm	NO_2_ (2.5 ppm)	130%
Varghese, O.K., *et al.* [13]	TiO_2_ nanotube arrays	46 nm 76 nm	H_2_ (1,000 ppm)	1,000% 90%

The current study introduces and develops the TiO_2_ nanotube array sensor, which is then used to conduct laboratory research testing of SF_6_ decomposition products and gas sensor tests on one of the SF_6_ decomposition components, SO_2_. The findings indicate that the sensor has good sensitivity and rapid response.

## 2. Experimental Section

### 2.1. Preparation of the TiO_2_ Nanotube Array

The present study generated a TiO_2_ nanotube array by an anodic oxidation method using a two-electrode system. A platinum metal piece was used as cathode, whereas a titanium piece was used as anode. The experimental processes are as follows: first, 0.5 mm thick Ti foil (area of 0.8 cm × 2.0 cm and purity of 99.94%) was burnished with sandpaper, soaked in 30% HCl solution, and heated to 80 °C for 20 min to remove the surface oxidation layer. Then, the Ti pieces were cleaned by washing with deionized water. The clean Ti pieces acted as the anode, whereas the platinum pieces acted as the cathode in the two-electrode electrochemical electrolysis pool. Between the two electrodes, a constant 20 V of anodic oxidation was applied continuously for 2 h. The electrolyte concentration was 0.1 M HF solution, and the electrolyte pH value was tested using a Model 3000 pH meter. Magnetic stirring was applied to ensure the uniformity of the Ti electrode surface electric current and temperature in the oxidation process and reduce the influence of the double electric layer between the electrolyte and electrode interface. After the reaction, the TiO_2_ nanotube array was cleaned by washing with deionized water, and dried in air heated from 2 °C/min to 500 °C in a muffle furnace for 1 h, and then removed after the temperature dropped to room temperature.

### 2.2. Production of TiO_2_ Nanotube Array

**Figure 1.**
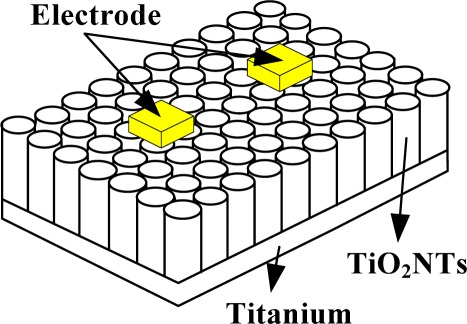
Structure sketch of the TiO_2_ nanotube array sensor.

A gas-sensitive element of the TiO_2_ nanotube array is different from a traditional gas-sensitive element. A TiO_2_ nanotube array is generated directly on a metal titanium piece, not coated on traditional Si or A1_2_O_3_ substrates. In the present study, a conductive silver glue was applied directly to create an electrical contact on the TiO_2_ nanotube array surface. The electrodes and TiO_2_ nanotube array were pasted firmly together to connect the leads. A schematic diagram of the sensor structure is shown in Figure 1.

### 2.3. TiO_2_ Nanotube Array Sensor Test Device and Method

**Figure 2.**
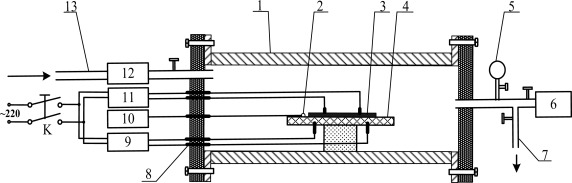
Detection test device for the TiO_2_ nanotube array sensor response measurement of SF_6_ decomposition products.

Figure 2 presents the detection test device for the TiO_2_ nanotube array sensor response measurement of the SF_6_ decomposition products. Shown in the figure are the following: 1. quartz glass tube; 2. thermal resistance probe; 3. carbon nanotube sensors; 4. ceramic heating slices; 5. vacuum form; 6. vacuum pump; 7. vent ducts; 8. terminals; 9. AC regulator; 10. temperature display apparatus; 11. impedance analyzer; 12. gas flow meter; and 13. inlet ducts.

In the current experiment, the standard gas containing the SF_6_ decomposition products that needs to be measured was injected through the inlet. The gas flow meter controlled and detected the measured gas flow rate. The ceramic heater and heat resistance of the probe controlled and measured the sensor surface temperature. The TiO_2_ nanotube array sensor was placed in an airtight quartz glass tube. The sensor detects the characteristics of the sensor resistance, and records the resistance of the whole process through the impedance analyzer. The relative variation of the resistance of the TiO_2_ nanotube array sensor (*i.e.*, sensitivity) was calculated using the following formula:

$$R\%=(R-R_{0})/R_{0}\times 100\%$$

where *R* is the sensor resistance after the injection of detected gas and *R_0_* is resistance in N_2_.

The response time of the sensor is the same as 90% of the amount of time that its resistance changes to the maximum amount. The TiO_2_ nanotube array has adsorption effects with the oxygen in air and water vapor; hence, to eliminate those factors, this experiment used the dynamic method [13]. The specific steps are as follows: before the sensitivity response test, high-purity N_2_ was first injected at a flow rate of 0.1 L/min, and at the same time, connected to the heating power supply. The voltage regulator was adjusted to control the surface temperature of the sensor (required to maintain a certain temperature) until the TiO_2_ nanotube sensor array resistance was stable. The value obtained for *R_0_* was recorded.

Second, one of the SF_6_ gas decomposition products, namely SO_2_, was passed, and the gas flow velocity in the device was maintained (the same as the previous N_2_ gas flow velocity). At this time, the sensor resistance exhibited pronounced changes and achieved stability (waves near one resistance) immediately. In the process, the resistance value *R* was recorded. Finally, when the sensor resistance was stable, high-purity N_2_ was injected again at 0.1 L/min velocity, until the resistance of the sensor gradually achieved numerical stability.

## 3. Results and Discussion

### 3.1. Morphology of the TiO_2_ Nanotube Array Obtained through Characterization and Analysis

**Figure 3.**
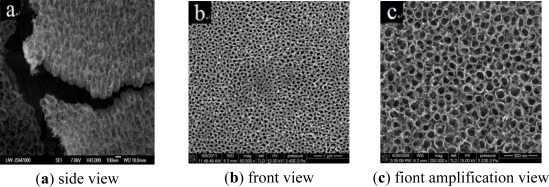
SEM images of the TiO_2_ nanotube array.

The sample was observed under a scanning electron microscope (SEM). In the present experiment, a JEOL JSM-7000 field emission SEM (Japan) was used. As observed from the SEM images, the anodic oxidation method and the above experimental scheme can grow a TiO_2_ nanotube array with a high order and directional growth, whose pipe diameter is about 80 nm and length of about 300 nm (shown in Figure 3).

**Figure 4.**
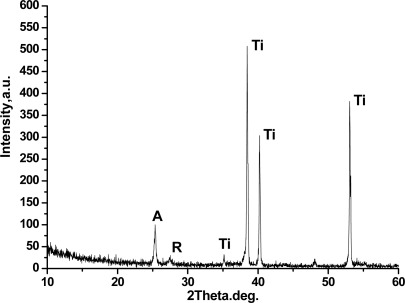
X-ray diffraction pattern of the TiO_2_ nanotube array.

Figure 4 shows an X-ray diffraction spectrum diagram of the TiO_2_ nanotube array. From the figure, the crystal face peak of strong anatase (A in the figure) exists at 2θ = 25.3°, and the 101 crystal face peak of weak rutile exists at 2θ = 27.4° (R in the figure). These findings indicate that the TiO_2_ nanotube array is mainly anatase, and a small amount of rutile phase is observed.

### 3.2. Influence of Working Temperature on the Gas-Sensitive Characteristics of the TiO_2_ Nanotube Array Sensor

The performance of metal oxide semiconductor gas-sensitive materials is greatly influenced by the working temperature. The present study tested the SO_2_ gas sensor response curve of the TiO_2_ nanotube array sensor at different working temperatures.

The prepared sensor was placed inside the mentioned test device (Figure 2). Through the temperature control device, the surface of the sensor was heated, and its surface temperature was controlled. In the current study, the gas-sensitive characteristics of the TiO_2_ nanotube array sensor were tested with 50 ppm SO_2_ at surface temperatures ranging from 20 °C to 400 °C.

**Figure 5.**
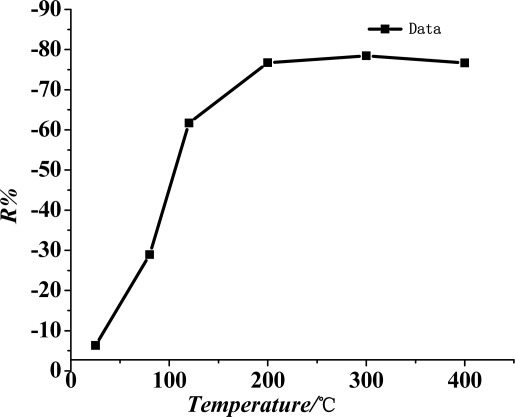
Sensitivity of the TiO_2_ nanotube array sensor at different working temperatures.

Figure 5 shows the curve of the sensitivity of the TiO_2_ nanotube array sensor at different working temperatures (*i.e.*, surface temperature). The chart indicates that when work temperature is lower, the sensitivity of the sensor increases with the rise in its working temperature. When the temperature reaches 200 °C, the sensor reaches it maximum sensitivity at −76%. When the working temperature continues to rise, the sensitivity tends to be saturated and remains basically unchanged. Therefore, the best working temperature for the TiO_2_ nanotube array sensor is about 200 °C.

**Figure 6.**
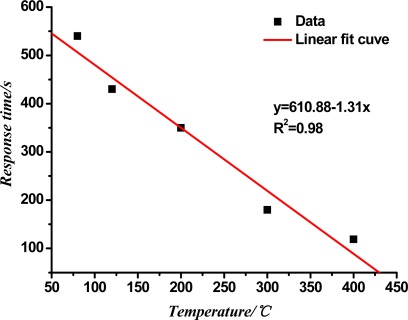
Response time of the TiO_2_ nanotube array sensor at different working temperatures.

Figure 6 shows the curve of the response time of the TiO_2_ nanotube array sensor at different working temperatures. In the figure, the response time of the sensor decreases with the rise in the working temperature, and has a certain linear relationship with the temperature. Through the linear fit, the linear correlation coefficient R^2^ is 0.98.

The molecular motion and diffusion of the gas speed up because of the increase in temperature, and the gas absorption and the dissociation rate of the sensor's surface increase so the sensor response time decreases with the increasing temperature.

### 3.3. Sensor Response of the TiO_2_ Nanotube Array to Different SO_2_ Concentrations

**Figure 7.**
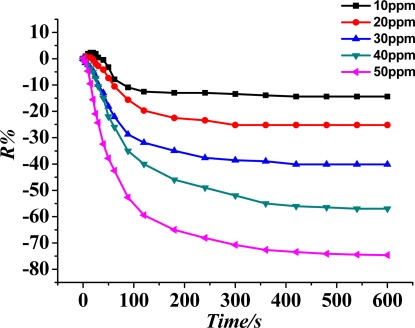
TiO_2_ nanotube array sensor response to different concentrations of SO_2_ at a 200 °C working temperature.

According to procedures of the experiment described in Section 2.3, under the condition that the sensor is under a 200 °C working temperature, the gas sensitivity (response curve) of the TiO_2_ nanotube array sensor is measured at SO_2_ gas concentrations of 10, 20, 30, 40 and 50 ppm, and the result is shown in Figure 7. The figure illustrates that the bigger the concentration of SO_2_ gas, the higher the sensor response (sensitivity).

**Figure 8.**
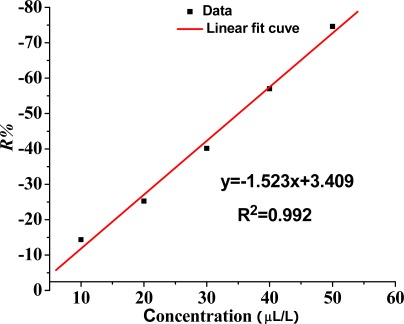
Linear relationship between the concentration of SO_2_ and the response of the TiO_2_ nanotube array sensor.

Therefore, based on the linear fitting, the relation between the concentration of SO_2_ gas and sensor sensitivity is a certain linear relation under a low concentration, and the linear correlation coefficient R^2^ is 0.992. As shown in Figure 8, the ability of the sensor to detect low concentrations of SO_2_ gas has a certain practical value.

### 3.4. Sensor Selectivity of the TiO_2_ Nanotube Array to Different SF_6_ Decomposition Components

**Figure 9.**
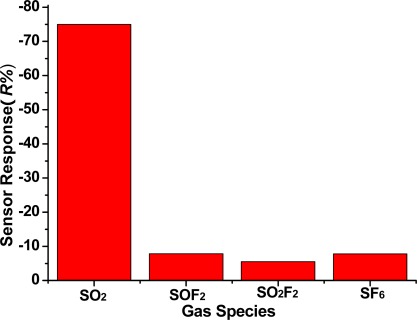
Sensor response of the TiO_2_ nanotube array to different SF_6_ decomposition components.

SO_2_ is produced by SF_6_ decomposition. It is necessary to study the TiO_2_ nanotubes sensor response to SF_6_ gas decomposition products and background gas SF_6_. According to methods and procedures of the experiment in Section 2.3, under the condition that the sensor is under a 200 °C working temperature, the gas sensitivity (response curve) of the TiO_2_ nanotube array sensor is measured at 50 ppm SO_2_, 50 ppm SOF_2_, 50 ppm SO_2_F_2_ and 99.999% SF_6_, and the result is shown in Figure 9. We can see that the sensor responses to 50 ppm SO_2_, 50 ppm SOF_2_, 50 ppm SO_2_F_2_ and 99.999% SF_6_ are −76%, −7.8%, −5.5% and −7.7%, respectively. This illustrates that the TiO_2_ nanotubes sensor has good selectivity for SO_2_ gas. It is suitable for checking SO_2_ gas, the main component of SF_6_ decomposition in the GIS.

### 3.5. Sensor Stability Testing of the TiO_2_ Nanotube Array

**Figure 10.**
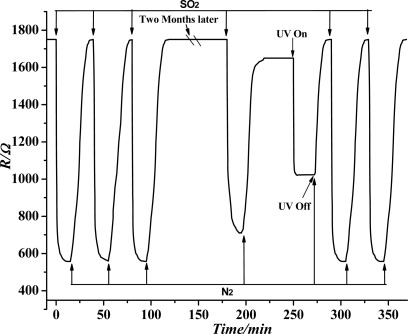
TiO_2_ nanotube array sensor stability testing curve.

According to the experimental method and process in Section 2.3, when the temperature was at 200 °C, the sensor stability test towards the SF6 decomposition component SO_2_ was conducted. The test results are shown in Figure 10. Before the sensor stability test, the TiO_2_ nanotube array sensor resistance remained basically unchanged when flowing pure N_2_ was injected. When injected with 50 ppm SO_2_ gas, the sensor resistance changed dramatically and achieved stability immediately. When injected with N_2_ after a certain period, the sensor resistance gradually returned to the initial value. The above experiment was tested three times, and we found that the sensor response of SO_2_ gas is invariable, and every time through nitrogen cleaning, the resistance of sensor can be returned to the initial value. Thus it is shown that the sensor has good stability. The sensor was tested agian after two months, when we repeated the 50 ppm SO_2_ gas experiment once again, and found that the sensor response falls, and resistance can’t return to the initial value, showing that the sensor has experienced chemical poisoning. Ultraviolet light is used for illumination and quick reduction of the resistance of the sensor. When stability is achieved, the resistance becomes smaller than the initial one. By passing nitrogen gas again, the sensor resistance increases gradually, and finally achieves stability. The sensor resistance returns to the initial value; when we performed the 50 ppm SO_2_ gas experiment two times, the sensor response reaches the level of the two months ago. That is, through the ultraviolet irradiation, the SO_2_ gas molecules adsorbed on the sensor can be washed away completely. Therefore, the TiO_2_ nanotube array achieves complete desorption results.

### 3.6. Discussion on the Mechanism of TiO_2_ Nanotube Sensor Array Gas-Sensitive Response

TiO_2_ as a sensitive material that detects gases through changes in physical properties, such as electric conductivity, when the gas comes in contact with a TiO_2_ molecule surface. Oxygen has a very strong adsorption. Oxygen in the air at room temperature adsorbs physically on the TiO_2_ surface. When a certain energy is gained, oxygen adsorbs on the TiO_2_ nanotube array sensor surface in the form of chemical adsorption. The common forms of chemical absorption oxygen are O_2ads_^−^, O_ads_^−^, and O_ads_^2−^, which relate to the environmental temperature [15]. The experimental results indicate that under low temperatures, the oxide surface exists in the form of a “molecular ion” O_2ads_^−^, and changes into a form of an “atomic ion” O_ads_^−^ and O_ads_^2−^ with the rise in temperature. At more than 450 K, O_ads_^−^ dominates the surface oxygen adsorption. In the current study, the working temperature of the sensor is 200 °C (473.15 K); hence, in the process, the oxygen in the air captures the surface electron of TiO_2_, changing into chemical adsorption. The chemical reaction equation is:

$$O_{2}+2e^{\prime }\to 2\;\;O_{\text{ads}}{}^{-}$$

When the sensor surface has reduced the SO_2_ gas, the response between the adsorption of oxygen and the SO_2_ gas sensor is as follows:

$${\text{SO}}_{2}+O_{\text{ads}}{}^{-}\to {\text{SO}}_{2}\;\;O_{\text{ads}}+e^{\prime }$$

SO_2_ gas undergoes an ionic reaction with the surface adsorption oxygen, removes an electron, releases back into the conduction band, and causes the conductivity of the TiO_2_ materials to increase, thus causing the resistance to decrease. In this manner, the TiO_2_ nanotube sensor plays a sensing function.

This finding is consistent with the experimental results. Although the TiO_2_ nanotube array and the TiO_2_ thin film semiconductor are different in microstructure and form, the above semiconductor adsorption mechanism is suitable for explaining the gas sensor response of the TiO_2_ nanotube array.

As seen in Figure 10, after two months, we repeated the 50 ppm SO_2_ gas experiment once again, and found that the sensor response was reduced, and the resistance can't return to the initial value. This phenomenon is due to the residual thermal decomposition of SO_2_ molecules fixed in the TiO_2_ nanotube arrays as the result of chemical adsorption. The adsorption energy of chemical adsorption is much larger than the physical adsorption capacity; hence, pure nitrogen gas flushing the sensor and low-temperature heating are not sufficient to remove completely the SO_2_ molecules left on the sensor by chemical adsorption. Therefore, ultraviolet light is used to desorb SO_2_ molecules attached on the sensor. In this manner, the sensor repeatability and service life can be improved because the forbidden bandwidth of the ultraviolet photon energy is almost the same as that of many metal oxide semiconductors. Hence, ultraviolet radiation can be absorbed effectively by the TiO_2_ nanotube array. The surface of the film and the inner portion undergo a range of physical and chemical processes. In the case of gas adsorption, ultraviolet radiation can be absorbed by the TiO_2_ nanotube array through electron-hole pair excitation, thus increasing the carrier concentration and reducing the grain interface barrier. Through these processes, the TiO_2_ nanotube array conductivity can be increased, and the resistor reduced. Ultraviolet radiation can be absorbed directly by gas molecules to produce desorption or stimulate chemical reactions between different types of molecules [16]. This finding indicates that the irradiation of ultraviolet light sensor can remove the remaining SO_2_ molecules effectively and thoroughly. This method can improve sensor repeatability and reduce sensor chemical poisoning, thereby increasing the service life of the sensor.

## 4. Conclusions

The current study is the first to report on the gas-sensitive characteristics of the TiO_2_ nanotube array of three-dimensional materials for the SF_6_ gas decomposition product SO_2_ gas. The following conclusions are drawn:

1. A highly ordered TiO_2_ nanotube array with directional growth is successfully produced by an anodic oxidation method. The TiO_2_ nanotube sensor array working temperature influences the sensitivity and the response time, and the best results are obtained when the sensor is at a working temperature of 200 degrees or so.
2. The current study analyzed the gas-sensitive characteristics of the TiO_2_ nanotube array of three-dimensional materials for the SF_6_ gas decomposition product SO_2_ gas. The results indicate that SO_2_ gas at low concentrations (10–50 ppm) creates a relative change in the resistance of TiO_2_ nanotube array, and that the response of TiO2 nanotube array sensor has a linear relationship with SO_2_ gas concentrations.
3. The irradiation of the sensor with ultraviolet light can remove the remaining SO_2_ molecules effectively and thoroughly, which can hasten the desorption process, thus avoiding chemical poisoning and extending service life.
